# Nanosphere Lithography on Fiber: Towards Engineered Lab-On-Fiber SERS Optrodes

**DOI:** 10.3390/s18030680

**Published:** 2018-02-25

**Authors:** Giuseppe Quero, Gianluigi Zito, Stefano Managò, Francesco Galeotti, Marco Pisco, Anna Chiara De Luca, Andrea Cusano

**Affiliations:** 1 Optoelectronic Division-Engineering Department, University of Sannio, 82100 Benevento, Italy; giuquero@unisannio.it (G.Q.); pisco@unisannio.it (M.P.); 2 Institute of Protein Biochemistry, National Research Council, 80131 Napoli, Italy; g.zito@ibp.cnr.it (G.Z.); s.manago@ibp.cnr.it (S.M.); 3 Institute for Macromolecular Studies, National Research Council, 20133 Milan, Italy; galeotti@ismac.cnr.it

**Keywords:** optical fiber sensor, optrode, lab-on-fiber, SERS, self-assembly, nanosphere lithography

## Abstract

In this paper we report on the engineering of repeatable surface enhanced Raman scattering (SERS) optical fiber sensor devices (optrodes), as realized through nanosphere lithography. The Lab-on-Fiber SERS optrode consists of polystyrene nanospheres in a close-packed arrays configuration covered by a thin film of gold on the optical fiber tip. The SERS surfaces were fabricated by using a nanosphere lithography approach that is already demonstrated as able to produce highly repeatable patterns on the fiber tip. In order to engineer and optimize the SERS probes, we first evaluated and compared the SERS performances in terms of Enhancement Factor (EF) pertaining to different patterns with different nanosphere diameters and gold thicknesses. To this aim, the EF of SERS surfaces with a pitch of 500, 750 and 1000 nm, and gold films of 20, 30 and 40 nm have been retrieved, adopting the SERS signal of a monolayer of biphenyl-4-thiol (BPT) as a reliable benchmark. The analysis allowed us to identify of the most promising SERS platform: for the samples with nanospheres diameter of 500 nm and gold thickness of 30 nm, we measured values of EF of 4 × 10^5^, which is comparable with state-of-the-art SERS EF achievable with highly performing colloidal gold nanoparticles. The reproducibility of the SERS enhancement was thoroughly evaluated. In particular, the SERS intensity revealed intra-sample (i.e., between different spatial regions of a selected substrate) and inter-sample (i.e., between regions of different substrates) repeatability, with a relative standard deviation lower than 9 and 15%, respectively. Finally, in order to determine the most suitable optical fiber probe, in terms of excitation/collection efficiency and Raman background, we selected several commercially available optical fibers and tested them with a BPT solution used as benchmark. A fiber probe with a pure silica core of 200 µm diameter and high numerical aperture (i.e., 0.5) was found to be the most promising fiber platform, providing the best trade-off between high excitation/collection efficiency and low background. This work, thus, poses the basis for realizing reproducible and engineered Lab-on-Fiber SERS optrodes for in-situ trace detection directed toward highly advanced in vivo sensing.

## 1. Introduction

Raman microscopy (RM) is a non-invasive vibrational spectroscopic technique that takes advantage of the inelastic scattering of light by vibrating molecules [1]. The characteristic bands in Raman spectra are narrow, easy to resolve, and specific to molecular structure, conformation, environment, and interactions with other molecules. RM measurements can be taken without labelling, and enable mapping the chemical heterogeneity of a specimen [2,3,4,5,6,7]. Additionally, its non-invasive and label-free nature is highly valuable for in vivo imaging [8,9]. The combination of RM and remote sensing through optical fibers realizes the *optrode* paradigm, which gives rise to an integrated and multiplexed sensing/imaging system for advanced biomedical applications [10,11,12]. Remote Raman sensing in optrode configuration has been validated in important clinical fields like brain surgery [13].

However, a crucial issue of Raman spectroscopy/imaging is the inherently weak nature of the light scattering signal, which dramatically undermines the sensitivity of the technique. A number of approaches can be taken to enhance Raman signals and reduce acquisition times, in order to increase the usefulness of this technique for clinical application [14]. A huge amplification of Raman signals can be achieved using suitable metallic nanostructures, which display a remarkable amplification of the electromagnetic field in their nanoscale proximity. The amplification is produced by electronic excitations termed localized surface plasmon-polariton resonances (LSPRs). These are at heart of the technique known as surface enhanced Raman scattering (SERS) spectroscopy. SERS enables detecting and resolving the chemical signature of even single molecules by concentrating the light down to the molecular scale. This has prompted a plethora of SERS applications for molecular sensing in chemistry, biology, and medicine [15].

Enhancing and expanding the Raman optrode protocol with SERS is an important aim that will establish a fundamental innovation in the field of medical diagnosis and therapy. SERS has the potential to provide fast clinical tissue imaging with the superior throughput necessary for in vivo application.

However, the implementation of a SERS optrode involves several challenges, pertaining to the fabrication of the SERS active surface on the optical fiber end facet [16]. The optical fiber tip is an unconventional substrate for the most “conventional” fabrication routes, which then cannot be easily adopted [17]. As matter of fact, effective approaches for fabrication of SERS surfaces on the fiber tip essentially were based on the metallization of roughened surfaces on fiber tips [18,19], or on the deposition of nanoparticle films on the fiber tip [20], rather than on the development of real lithographic processes. As a consequence, the absence of regular patterns limited the reproducibility of the fabricated probes, while the ability to obtain regular and repeatable patterns on the optical fiber tip is essential to obtain highly efficient substrates for quantitative SERS measurements.

Recently the development of SERS probes with optical fiber technology has been strongly revitalized, thanks to the significant growth in the field of nanotechnology and to the possibility of producing well-ordered nanopatterns directly on optical fiber tip. All these efforts, often named “Lab-on-Fiber” technologies [21,22], opened new opportunities for the implementation of several laboratory functions on the fiber tip by developing specific routes for using optical fibers as an exclusive substrate for micro- and nanotechnologies. For example, Kostovski et al. used nanoimprint lithography to produce a silver nanostructure array (as a SERS surface) directly onto the end faces of standard silica optical fibers by replicating the pattern of cicada wings [23]. Smythe et al. built a SERS optrode by fabricating a nanoantenna array on a planar substrate using electron beam lithography and then transferring it to the fiber tip [24]. Bouvrée et al. used a multi-step process based upon e-beam lithography to create regular patterns (concentric rings or lines) on the optical fiber tip where gold nanoparticles are then immobilized [25]. Andrade et al. designed and fabricated SERS surfaces composed of both circular and bow tie-shaped gold nanohole arrays by using the direct FIB milling of a gold layer deposited on the single mode fiber tip [26].

In all these studies, remarkable precision was achieved in defining the pattern on the fiber tip, and valuable performances were attained by confirming the strong potential of fiber optic SERS optrodes. Major details on these achievements can be found in recently published reviews involving Lab on tip devices for SERS applications [21,22,27]. Nevertheless, in spite of increasing applicability and research interest, SERS optrodes are still largely confined to specialized research laboratories. 

One major obstacle in the way of practical establishment of SERS probes has been the challenge of fabricating stable, reproducible and sensitive SERS substrates on the optical fiber tip by using a low-cost process [16]. Additionally, use of the optical fiber as a waveguide leads inherently to specific issues such as the background due to the fluorescence and Raman scattering associated with the optical fiber constituent materials, limiting the SERS optrode performances.

With regard to the difficulty of obtaining reproducible SERS active surfaces on the optical fiber tips, self-assembly processes could supply a significant technological contribution by granting low fabrication costs and high throughput. Alongside the aforementioned technological advancements of using conventional lithographic approaches on the optical fiber tip, developments in self-assembly techniques have led to interesting and innovative fabrication approaches for SERS optrodes, such as the deposition of silver nanoparticles by means of a layer-by-layer approach [28], or the deposition of metallic clusters of nanoparticles by electrostatic assembly [29].

On the other hand, selection of the optical fiber is critical and its coupling with the Raman spectrometer should be carefully taken into account in order to improve the collection efficiency and reduce the background in the Raman spectra [30,31]. Recent studies suggested that an ideal fiber probe should have low dopant concentration in the fiber core, high numerical aperture (NA), and small core size [31], in order to reduce the background and to maximize both the collection efficiency and the coupling between the fiber and the Raman spectrometer. Nonetheless, these parameters are not independent and the “*a priori*” identification of the optimum configuration among different commercial fiber probes is not trivial. More complex solutions, involving the design and the use of specialty fibers [32], rare- earth doped fibers [33] or single crystal sapphire fibers [34,35,36], were also investigated to provide a low Raman background signal [37].

Recently we demonstrated the development and engineering of lithographic processes based on self-assembly phenomena, and created metallo-dielectric patterns on the optical fiber tip [38,39,40,41]. Additionally, we demonstrated the capability of obtaining regular and repeatable SERS active surfaces on the fiber tip (via colloidal self-assembly) in the form of close-packed arrays (CPAs) of nanospheres covered by thin films of gold [41]. The SERS properties of the proposed sensor were demonstrated with a common analyte—crystal violet—as a model substance [41]. However, our studies were mainly focused on assessment of the fabrication process for the realization of repeatable SERS probes.

In this work, starting from the promising results achieved in terms of reproducible metallo dielectric patterns on the optical fiber tip, we first investigated the SERS performances through the evaluation of the EF and its dependence on the pattern features (pitch and gold thickness). To this aim, we used the biphenylthiol (BPT) monolayer as benchmark analyte to estimate the enhancement factor (EF) of the fabricated SERS active surfaces. Performances and repeatability intra- and inter-sample have been assessed, allowing for selection of the most promising SERS template. Then, we selected several commercially available optical fibers and tested them with a BPT solution (1 mM) in order to identify the fiber platform which provides the best performances in terms of Raman excitation/collection efficiency and related background.

The overall results obtained in this study set the fundamental milestones for the realization of repeatable and engineered Lab on Fiber SERS optrodes for in vivo clinical studies.

## 2. Materials and Methods

### 2.1. SERS Substrate Fabrication by Nanosphere Lithography

**Figure 1:**
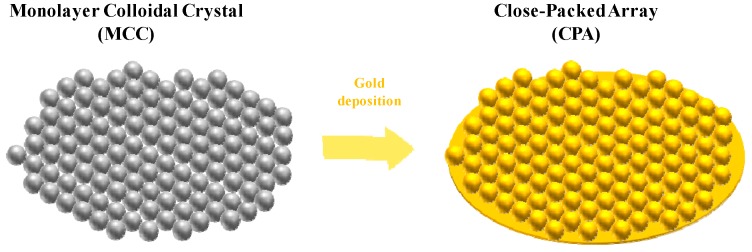
Schematic of the nanospheres substrate in close-packed array configuration.

The SERS active surfaces are composed of hexagonally CPA of nanospheres, covered by thin films of gold, as schematically shown in Figure 1. In order to measure the performance of the SERS surfaces, independently from the selected fiber probe, we fabricated the SERS samples in a planar configuration (on the coverslip slides, quartz CFQ-2557, UQG Optics, 150 μm thickness). The fabrication process follows the procedure described in ref. [41] and it is here only briefly recalled for sake of completeness.

The proposed approach essentially relies on the self-assembly of polystyrene (PS) nanospheres at the air-water interface. The PS nanospheres are commercially available in aqueous suspension (2.5% wt in water, Polysciences Europe GmbH, Hirschberg an der Bergstrasse, Germany). The concentration is adjusted by precipitation in a micro-ultracentrifuge and resuspension in different volumes of 1:1 ethanol/water. To optimize the assembly, the final wt concentrations are set as follows: 500 nm at 10%, 750 nm at 15%, and 1000 nm at 6%. Each nanosphere suspension is released with a microsyringe at the air water interface using a silicon conduit plate. After the monolayer colloidal crystal (MCC) is self-assembled at the water surface, it is transferred onto the planar substrate by dipping the desiderated substrate into the water bath and lifting it towards the floating MCC island. The gold layer is successively deposited on the MCC by thermal evaporation. The flat substrate is coated with a MCC of spheres with three different diameters (*d* = 500, 750, 1000 nm) and three gold layer thicknesses (*h* = 20, 30, 40 nm).

### 2.2. Spectral Characterizations

Spectral reflectance measurements were performed by illuminating the planar samples with a broadband optical source (covering the wavelength range 400–1000 nm) and redirecting the reflected light (via a-six-fibers standard reflection probe) to a spectrophotometer. The sample is placed on a motorized XYZ positioning stage. The measured reflectance spectrum of the sample is finally normalized to that of an aluminum mirror.

### 2.3. Biphenyl-4-Thiol Sample Preparation

Biphenyl-4-thiol 99% (Sigma-Aldrich, St. Louis, MO, USA) was dissolved in ethanol to achieve a final concentration of 1 mM for SERS measurements. The SERS substrates were soaked in the BPT solution for 24 h, then rinsed in pure ethanol repeatedly to remove free molecules. The formation of the BTP monolayer guarantees a uniform coverage of the gold surface with known packing density of approximately 2 molecules per nm^2^ [42]. The actual density depends on the specific molecule properties and can be even larger [43]. Conservatively, given the increased area provided by the patterned gold nanostructure with respect to the flat surface, we estimated an effective molecular density of about 4 molecules over nm^2^ [42]. A highly uniform self-assembled monolayer of BPT was obtained and used for the SERS experiments.

A cuvette containing 2 mL of 100-mM BPT solution was used for conventional spontaneous Raman experiments which were to determine the detection efficiency through the optical fiber. As such, Raman spectra were acquired with both the optical fiber coupled to the Raman microscope with the tip immersed directly in the cuvette, and by using the microscope with same optical configuration (objective, slit, pinhole, power, integration time) but without optical fiber delivery.

### 2.4. Raman and SERS Measurements

Raman and SERS spectra were recorded using an inverted confocal Raman microscope (XploRA INV, Horiba Jobin Yvon, Villeneuve d'Ascq, France) [8], equipped with a 785 nm wavelength diode laser. 

For the SERS experiments (planar configuration), a 60× water immersion objective (Ti-2000 Eclipse, NA = 1.2, Nikon Instruments Europe BV, Amsterdam, The Netherlands) was used to focus the laser beam onto the sample (the beam waist was about 2 μm) and collect the scattered light. The back scattered light from the sample was spectrally filtered by a holographic notch filter and focused onto the spectrometer entrance slit (set at 100 μm). The spectrometer was equipped with an 1800 lines/mm holographic grating. Finally, the SERS signal was detected by a thermoelectrically cooled CCD camera. Each SERS spectrum, acquired with an integration time of 1 s, and a laser power on the sample of about 2.5 mW, was background corrected with polynomial fit to the fourth degree. 

In the intra-sample reproducibility study, for each substrate a set of 49 SERS spectra was acquired (using 1 s of exposure time for each spectrum) from a square grid of 7 × 7 points, with a step between adjacent points of about 7 µm, yielding a small map. Maps were recorded by plotting the distributions of the SERS intensities of the BPT band around 1590 cm^−1^ and repeated at 3 random locations on the sample. The intra-sample relative standard deviation (RSD) was measured as the variability of each individual spectra after background correction with the average spectra.

**Figure 2:**
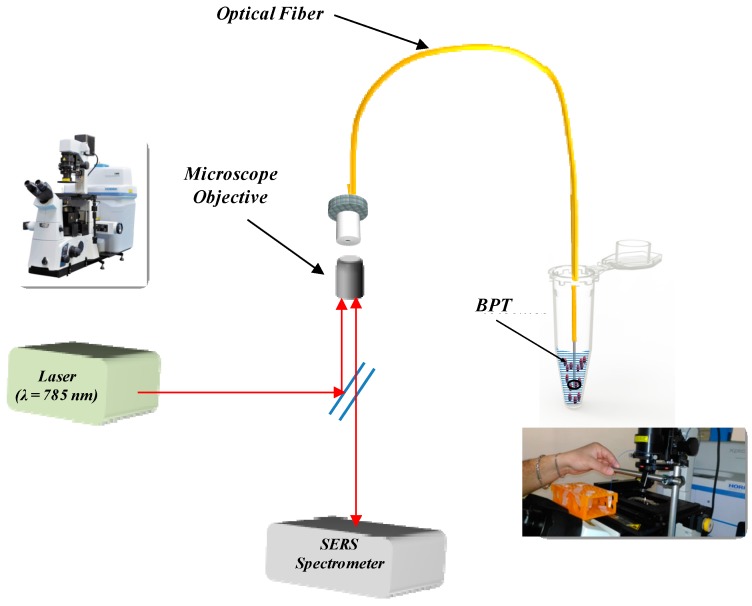
Schematic of the experimental setup for the optical fiber type selection composed by the Xplora Raman microscope (with relative picture) and the optical fiber immersed in the BFT solution (with related picture).

For Raman experiments, the Raman spectrometer was coupled to the optical fibers, as schematically displayed in Figure 2. A 10× objective (Nikon S Fluor, NA = 0.5) matching the NA of the optical fiber was used to guide the laser beam onto the sample and collect the backscattered light. The spectrometer slit was set at 100 µm to have high spectral resolution of 1.5 cm^−1^. With this configuration, the ellipsoidal collection volume was measured with a knife-edge technique at the Raman frequency of silicon (514 cm^−1^) and gave a diameter of 16 µm and a Rayleigh length of about 200 µm. Each Raman spectrum was acquired with an integration time of 10 s and laser power on the sample of 25 mW.

### 2.5. Fiber Probe Selection

The main specifications of the selected fibers, according to the information available in their datasheets, are listed in Table 1. As reported in the table, all the optical fibers are multimode fibers with pure silica cores of low hydroxyl content. The fibers operate in the visible wavelength range in order to allow the propagation of both the excitation and scattered light. The selected fibers differ in core diameter (ranging from 50 up to 400 µm) and have different cladding materials (fluorine-doped silica or hard polymer), resulting in various NAs (from 0.22 up to 0.5).

Each fiber section was preliminarily prepared starting from a commercial patch-cord ending with ceramic ferules (with external diameter of 2.5 mm) at both sides. The terminal end having the ceramic ferule was positioned and centred under the microscope objective at the focal plane, while the other side was prepared by stripping and carefully cleaving the fiber end to have an overall fiber section 40 cm long. After cleaving the terminal end of each fiber, the fiber tip was inspected under the microscope to ascertain the cut regularity. The cleaved end was immersed in a 1.5 mL micro-centrifuge tube containing the BPT solution (100 mM).

## 3. Results and Discussion

### 3.1. SERS Structure Performances

**Figure 3:**
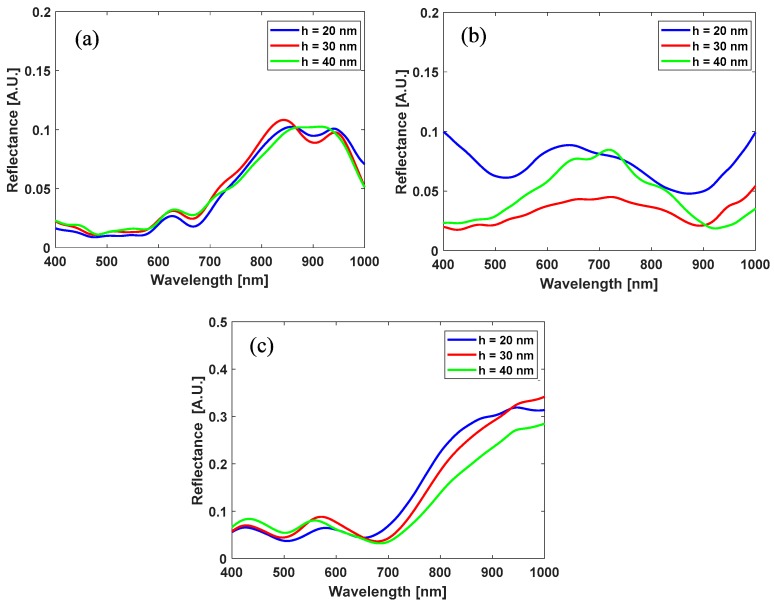
Reflectance spectra measured on CPA substrates with different diameters (*d* = 500 nm (**a**), 750 nm (**b**), 1000 nm (**c**)) and different gold films (*h* = 20 nm, 30 nm, 40 nm).

Before SERS enhancement evaluation, the LSPR spectral response was investigated as a function of the geometric parameters by measuring the reflectance spectra of the structures, i.e., the far-field light spectrum back-scattered to the detector. As is clearly visible in Figure 3, the maximum reflectance is achieved at *d* = 500 nm, and in a spectral region which justifies Raman excitation at 785 nm. Of note, the reflectance spectra cannot be used to evaluate the effective near-field amplification experienced from molecules, which must be the subject of the SERS-substrate EF evaluation, as described below.

To analyze and compare the SERS activity of substrates with different geometries, in particular hexagonally closely packed arrays (CPAs) of PS nanospheres with differing diameters (*d* = 500, 750, 1000 nm) and differing gold films (*h* = 20, 30, 40 nm), we used the BPT monolayer as our Raman probe molecule. Indeed, the BPT molecules self-assemble on gold forming semi-covalent bonds with a definite packing morphology. A highly uniform BPT monolayer results on the gold surface, with a density of about 4 molecules over 1 nm^2^.

Non-structured flat gold films do not provide enough amplification to detect the SERS signals of the BPT monolayer, therefore the presence of the enhanced signal can be used to monitor the presence of hot-spots induced by the nanostructure morphology [48].

Additionally, once the laser beam parameters are characterized (beam waist of 2 μm), the number of molecules *N*_SERS_ in the laser scattering area probed during SERS measurements is precisely known (*N*_SERS_ = 5 × 10^7^), and can be used for estimating the *SERS-substrate* EF by using the equation:

EF = (*I*_SERS_/*N*_SERS_)/(*I*_RS_/*N*_RS_) (1)

where *I*_SERS_ and *I*_RS_ are the intensities of the band at 1590 cm^−1^ for the SERS and Raman spectra, and *N*_RS_ is the average number of the BPT molecules in the scattering volume for the Raman measurements. The scattering volume was characterized under the same optical configuration. The Raman signal was collected from a powder sample of BPT. From density and molecular weight, and the scattering volume, *N*_RS_ can be readily estimated [49].

**Figure 4:**
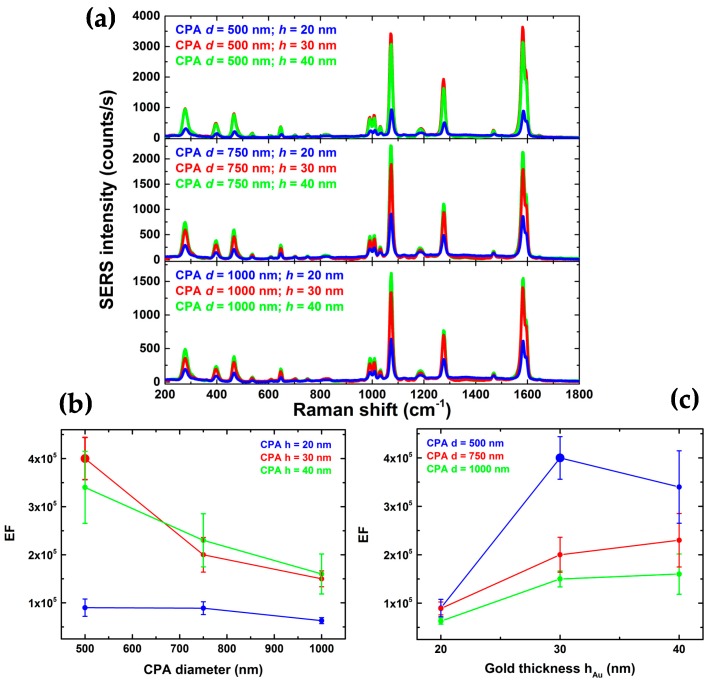
(**a**) Average BPT SERS spectra of 49 acquisitions, for the considered substrates: hexagonally closely packed arrays of PS nanospheres—CPA—with different diameters (*d* = 500, 750, 1000 nm), and different gold films (*h* = 20, 30, 40 nm). Measured enhancement factor for the different analysed geometry as a function of the CPA diameter (**b**) and gold layer thickness (**c**).

Average SERS spectra of the BPT monolayer for each substrate (CPA prepared with various bead diameters and gold films) are reported and compared in Figure 4a. The SERS spectrum of BPT presents several known differences from its spontaneous Raman version because of the change of molecular polarizability when the molecule is adsorbed to gold. This can then be used to determine the monolayer condition and hence the reliability of the molecular density estimation [48]. Based on our previous research about the SERS quality of the plasmonic substrates [15], the EF for different samples was estimated by comparing the BPT peak intensity at 1590 cm^−1^ and using the Equation (1). The highest measured EF = 4.0 × 10^5^ can be achieved with the CPA geometry with a PS diameter of 500 nm and a nominal gold thickness of 30 nm. The SERS contribution came from the “hot spots” of the nanogaps between the gold coated nanospheres [50]. Indeed, the CPA diameters and therefore, the geometry of the plasmonic structure should play an important role in the determination of the substrate performance at the analyzed wavelength (785 nm). However, by decreasing the bead diameter from 1000 nm to 500 nm, as shown in Figure 4b, only a slight increase of the SERS signal can be observed (factor 2). It is likely that a smaller bead size implies a denser structure of the hot spots on which the BPT molecules are adsorbed. We found that additional optimization can be performed by altering the thickness of the gold layer. We varied the layer from 20 to 40 nm (see Figure 4c), finding that the EF reaches an optimum at the nominal 30 nm gold layer (4× times better than the signal with 20 nm gold layer). In this case, within the area scanned by the laser spot, the number of hot spots should be homogenous and the SERS variation should depend on the geometry of the substrate. It is worth stressing that the SERS-substrate EF is not a measure of the strength of the hot-spots but a practical estimation of the near-field amplification averaged over the area of the nanostructure probed by the laser beam (the *single-molecule* EF can be larger even of two orders of magnitude at the hot-spots [49]). The spatially averaged EF which we have estimated is thus comparable with state-of-the-art SERS substrate EFs typically achievable with highly-enhancing gold nanoparticles, for which the highest EF is typically below 10^6^ [51]. Therefore, the value we have measured is highly promising for challenging sensing of biological materials like cell membranes or proteins in biological environments [52,53,54].

**Figure 5:**
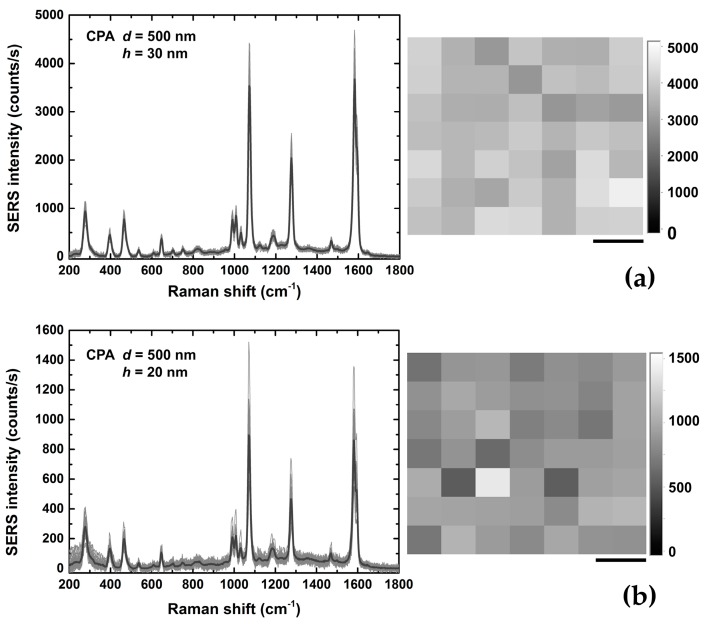
49 BPT SERS spectra (grey lines) and an intensity map of the band at 1590 cm^−1^ for two selected substrates: CPA *d* = 500 nm, gold thickness *h* = 30 nm (**a**) and *h* = 20 nm (**b**). The mean SERS spectrum (black line) is additionally shown. The intra-sample relative standard deviation was respectively about 9–10% for the substrate shown in (**a**) and about 20–22% for the substrate in (**b**). (scale bar = 10 μm).

The different SERS substrates were investigated in terms of intra- and inter-sample repeatability by mapping the intensity of the BPT band at 1590 cm^−1^. For all the substrates, by scanning the structure with the laser we were able to measure the enhanced BPT signals as a function of the spatial coordinates so as to determine quantitatively the spatial reproducibility of the structure. Figure 5 compares the BPT SERS map and relative spectra obtained on two different substrates: CPA *d* = 500 nm, *h* = 30 nm and CPA *d* = 500 nm, *h* = 20 nm. Within the area scanned by the laser beam, the two substrates should be uniform in terms of SERS hot spot distributions. However, the presence of small granularity for the substrate with a gold layer of 20 nm affects the signal intensity and repeatability. The Au substrates featuring the best repeatability are those developed using CPA with the bead size of 500 nm, in the presence of a layer of gold that is 30 nm-tick. This kind of substrate features an intra-sample RSD of about 9%. Mapping of three regions of each substrate and three replicates of each type of substrate was performed after sample incubation with BPT, and the inter-sample RSD for the sample CPA *d* = 500 nm and *h* = 30 nm was of about 12–15%. For ultrasensitive detection, the fluctuation of the SERS signal must be carefully evaluated because the signal may strongly depend on the hot spot density of the plasmonic architecture. Thus, reproducibility is an important parameter that must be assessed to enable quantitative SERS analysis. In this case, the SERS intensity can reliably provide a measurement of the analyte quantity, whereas its Raman fingerprint chemically identifies the species [52].

**Figure 6:**
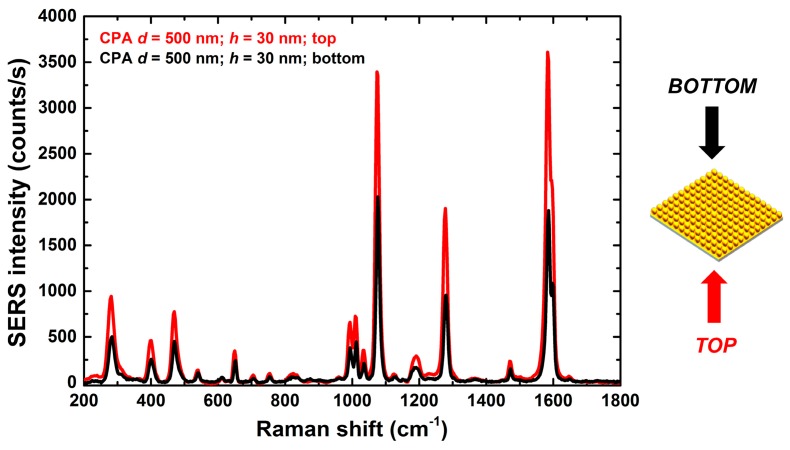
BPT SERS spectra, for the CPA substrate (*d* = 500 nm, gold thickness *h* = 30 nm), acquired in top and bottom configuration (sketch on the right).

Figure 6 compares the BPT SERS spectra obtained on the CPA substrate (*d* = 500 nm and *h* = 30 nm) in top and bottom configuration. These measurements were conducted in order to single out possible issues of the SERS substrate characteristics related to interrogation through the optical fiber versus interrogation in air. The top configuration corresponds to the optical fiber condition in which the laser light illuminates the glass, then the CPA gold substrate and finally the BPT deposited on top of it. In the bottom configuration, instead, the laser light in air hits the BPT deposited on the substrate first, then the CPA gold substrate on the bottom. As is evident, the specific asymmetric NP geometry favours backscattering towards the detector. In other words, not only does interrogation of the SERS substrate through the optical fiber not reduce the backscattering signal, but actually the optrode configuration (top) is the most favorable excitation configuration because of the *z*-asymmetric radiation pattern of the SERS substrate (z being the optical axis of the optical fiber).

### 3.2. Fiber Probe Performances

For the Raman analysis, we collected commercially available fibers featuring a core of pure silica and operating in the visible spectral range. Fiber selection was performed by taking into account the main outcomes of previous studies. We considered only those fibers with low dopant concentration in the fiber core, because it is recognized that a doped core supplies a higher background in the SERS spectrum with respect to pure silica fibers [30,31]. Furthermore, in order to maximize the collection, an efficiency coupling between the fiber and the Raman spectrometer system should be granted [30]. In this regard, the fiber should have a NA that is high for improving the collection of the scattered light, but lower than the microscope NA in order to have a good coupling. Similarly, the core diameter should be lower or equal than the slit width projection in the focal plane. Nevertheless, all these constraints, together with the interdependence of the fiber parameters, do not allow inference of the best fiber probe in terms of both low background level and high collection efficiency. Therefore, the optimum fiber probe, relatively to the Raman spectrometer system used, is determined empirically using the four fibers, listed in Table 1, with core diameter ranging from 50 up to 400 µm.

**Figure 7:**
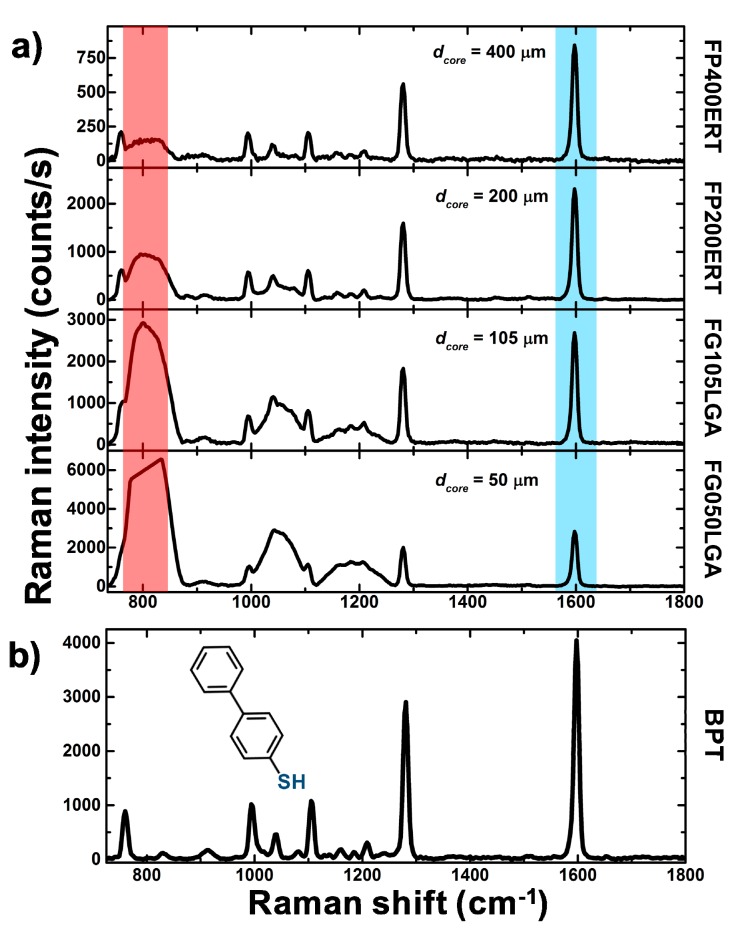
(**a**) Raman spectrum from 100 mM Biphenyl-4-thiol (BPT) solution through different optical fibers (**b**) Raman spectrum from the BPT solution with the Raman microscope without the fibers.

In Figure 7a, we show the Raman spectra, in the range between 750–1800 cm^−1^, obtained by illuminating the BPT solution (100 mM), and collecting the scattered light through the selected optical fiber probes. The Raman spectrum of the BPT solution acquired with an XploRA microscope (without fibers) is reported in Figure 7b for comparison. For all of the considered fibers, the BTP Raman bands can be easily recognized: the two strong bands at 1282 and 1590 cm^−1^ due to the ring ν(C=C) and four smaller peaks in the region 950–1100cm^−1^ due to δ (C-H) are visible [55]. Nonetheless, when the light goes through the fibers, additional Raman bands around 800, 1050 and 1200 cm^−1^ associated to silica are detectable in the fingerprint region [56,57]. Specifically, when we use the fiber FG050LGA and FG105LGA, the silica band at 800 cm^−1^ is particularly intense, obscuring the BPT peak due to a ν (S-C) around 757 cm^−1^. Similarly, the three BPT peaks in the wavenumber region 950–1100 cm^−1^ have an unfavourable signal-to-background ratio. Interestingly, when we use the fibers with larger core diameter (FP200ERT and FP400ERT) and larger NA the interference with the silica Raman bands decreases and even the small BPT band at 757 cm^−1^ becomes visible. The spectral region below 750 cm^−1^ is dominated by two broad silica bands (450 and 600 cm^−1^) due to the fiber core, and are therefore not shown. Additional considerations can be given by analysing the BPT bands at 1282 and 1590 cm^−1^, indeed the absolute intensities of these peaks increase with decreasing the fiber core diameter, reaching a maximum value of about 3000 counts/s with the 50 µm core size fiber. This trend can be explained in terms of a better collection efficiency achievable with smaller fiber cores [30]. When we use fibers with increasing core size, only a portion of the light back-propagating in the fiber probe fits within the projected area (i.e., onto the focal plane) that is seen by the whole collecting system (≈π R^2^ = π 50^2^ µm^2^). Consequently, the power exceeding this area is lost, without contributing to the Raman spectrum.

**Figure 8:**
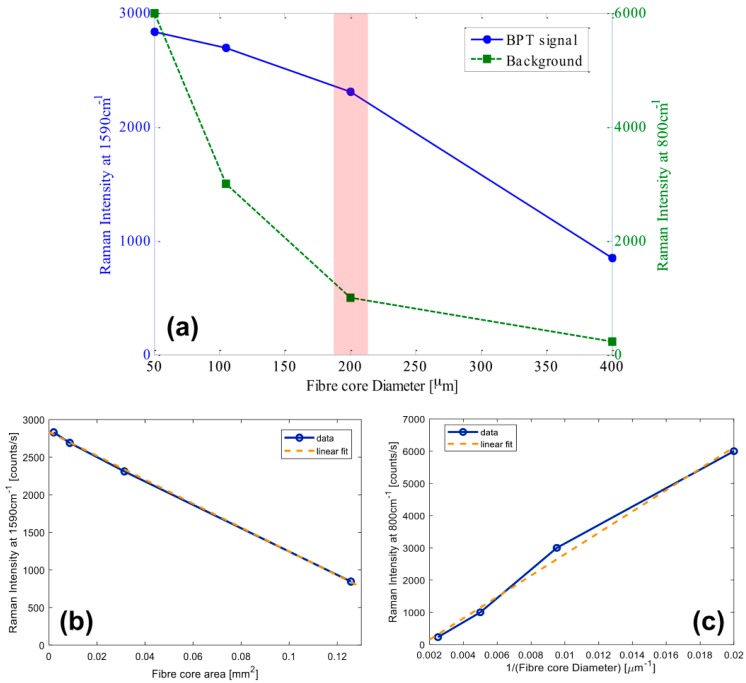
(**a**) Raman intensities for the band at 1590 cm^−1^ and at 800 cm^−1^, respectively versus the core diameter; (**b**) Raman intensity for the BPT band at 1590 cm^−1^ and linear fit versus fiber core area; (**c**) Raman intensity for the silica band at 800 cm^−1^ and linear fit versus the inverse of fiber core diameter.

However, to select parameters for the best performing fiber, both the intensity of the BPT band at 1590 cm^−1^, and the intensity of the broad silica background about 800 cm^−1^ should be considered. In Figure 8a, we display the BPT signal and background intensities versus the fiber core diameter. By increasing the fiber core diameter, the BPT signal decreases with a significant reduction of the background silica peaks. It is worth noting that both reductions do not follow the same behavior. More precisely, in Figure 8b,c, we can see that the BPT signal is decreasing linearly with the core area (i.e., with the square of the diameter), while the background intensity can be fairly well approximated with a linear reduction with the inverse of the diameter.

As a result, a good compromise between high intensity for the BPT fingerprint (which requires a small fiber core diameter) and a low silica background (which requires a fiber with large diameter) can be reached with the optical fiber FP200ERT, which maximizes the difference between the signal and the background (see Figure 8a).

## 4. Conclusions

In this work, we investigated the potential to engineer Lab-on-Fiber SERS optrodes in order to provide advanced and repeatable SERS substrates integrated onto the optical fiber tip. To this aim, we focused our attention on the nanosphere lithography approach, which allowed for the possibility of having repeatable SERS substrates on non-conventional platforms such as on the optical fiber tip. As a first but crucial step, we assessed the performances in terms of true EF pertaining to SERS substrates composed of hexagonal CPAs of PS nanospheres with different diameter (*d* = 500, 750, 1000 nm) and different gold film thickness (*h* = 20, 30, 40 nm) in order to select the most promising SERS pattern. A monolayer of BPT was used as a SERS benchmark to obtain a reliable estimation of the number of molecules contributing to the scattering signal, and thus correct evaluation of the related EF.

The different SERS substrates were investigated in terms of intra- and inter-sample repeatability by mapping the intensity of the BPT band at 1590 cm^−1^. By comparing our resultant performances, we found that the CPA geometry with a PS diameter of 500 nm and nominal gold thickness of 30 nm exhibited the highest EF (4.0 × 10^5^). The spatially averaged EF herein estimated is thus comparable with state-of-the-art SERS substrate EFs typically achievable with highly-enhancing gold nanoparticles, for which the highest EF is typically below 10^6^ [38]. Therefore, the value here measured is highly promising for challenging sensing of biological materials like cell membranes or proteins in biological environments [39,40,41]. Furthermore, these kinds of substrates feature a valuable intra-sample (RSD of about 9%) and an inter-sample (RSD of about 12–15%) repeatability, confirming the high level of reproducibility outlined by previous spectral and morphological investigations [41]. These excellent values of reproducibility suggest the possibility of *quantitative* SERS analysis, in which SERS intensity can reliably provide a measurement of the analyte quantity, whereas its Raman fingerprint chemically identifies the species [39]. The robustness of the substrate architecture readily enables applications of such SERS optrodes in a wide variety of relevant biochemical and biomedical application scenarios such as label-free bioassays based on Raman spectroscopy.

As a second important step towards engineering of the SERS optrode, Raman excitation/collection efficiency and related background pertaining to different commercially available optical fibers were investigated using a reference BPT solution as benchmark. This allowed us to identify the suitable optical fiber platform for Lab-on-Fiber SERS optrodes. We found that the excitation/collection efficiency linearly decreases with the fiber core area. This behaviour can be explained by considering that only a portion of the light back-propagating in the fiber probe fits within the projected area (i.e., onto the focal plane) seen by the collecting system. Consequently, the power exceeding this area is lost, without contributing to the collected Raman spectrum. Inversely, the spectral background due to the silica (specifically the broad band at 800 cm^−1^) increases linearly with the inverse of the fiber diameter. As a result, we selected the fiber probe with a core of pure silica, a 200 µm diameter, and a high NA (i.e., 0.5) because it offered the best trade-off between collection efficiency and background.

In conclusion, this study sets the required milestones for the realization of advanced and repeatable Lab-on-Fiber SERS optrodes as miniaturized sensing probes.

## Figures and Tables

**Table 1:** Optical fiber parameters.

Optical Fiber Type	Wavelength Range	Core Diameter	Optical Fiber Core Material	NA	Optical Fiber Cladding Material
FG050LGA [44]	400–2400 nm	50 µm	Pure silica (Low OH)	0.22	Fluorine-Doped Silica
FG105LCA [45]	400–2400 nm	105 µm	Pure silica (Low OH)	0.22	Fluorine-Doped Silica
FP200ERT [46]	400–2200 nm	200 µm	Pure silica (Low OH)	0.5	Hard Polymer
FP400ERT [47]	400–2200 nm	400 µm	Pure silica (Low OH)	0.5	Hard Polymer
